# Supplementary material for: Effects of Zinc Oxide Nanoparticles on Growth, Development, and Flavonoid Synthesis in Ginkgo biloba
Source: Int J Mol Sci. 2023 Oct 30;24(21):15775. doi: 10.3390/ijms242115775 (PMC10649971; doi:10.3390/ijms242115775)
Supplement: Supplementary file 1 [file ijms-24-15775-s001.zip › ijms-2662676-supplementary.pdf]

Table S1. Primer sequences for qRT-PCR and gene cloning

Primer ID	Forward primer sequences (5′–3′)	Reverse primer sequences (5′–3′)
GAPDH	CTGCCAAGGCTGTAGGTAAGG	TCAGATTCCTCCTTGATGGCG
Gb12934	TTCCACTGGTTCAACGGT	TGGTCATCAACTTCTACCCA
Gb02188	GAATTGGACATGACAGAGGGCAGAG	GAGTGGTAGAGATGAAGAGGCAAACG
Gb11301	TACACGGCTTACAATTACTCGGACTTG	CAGACCCACCTCTTCCTCTCTCATC
Gb16307	ATCCAGCCGAGTTGAATATGAGTGAAG	TTAAATCTGACTGCGGTGTCCATCC
Gb16632	GGAGGAAAGACGGAAAGTAGCAGATG	ATACCCACCAAGAAGAAGATCCAACAC
Gb30377	CCAGAGGCGATTCAGGGTATTGTTC	CGGCGACCAAAGATTGAAAGGATTC
Gb30412	GGACCTCGTTGGCGTATGCTTAG	CCATTCGGAACACCTGATCTCTTCTG
Gb32738	TCCTCCGCCAACTCCGTTAC	ACAGTGGAAACGGAGCCCAA
Gb14028	AGAGGTGACAGAAGAATACAGCAACAG	TTCAGCTCCATTTCCAGATTCTCATCC
Gb14031	ATGGCATCTCTCCCTCGCTCTTC	CAGGCATACACTTCCTTCTCCTCTTG
Gb24242	AAAGAAAGCCATAGAGTCAGTCACAGG	CTGAGCACATATAGCGGTCTTCCAC
Gb19002	CGAATTACCAACAGCGAGCACAAG	TCTCCTTCAGTATCTCCTCCGTCAAG
Gb12934(CDS)	ATGGTCCGCGTTCTTGTATTTC	CTGGCCGGAATGGAGGGG

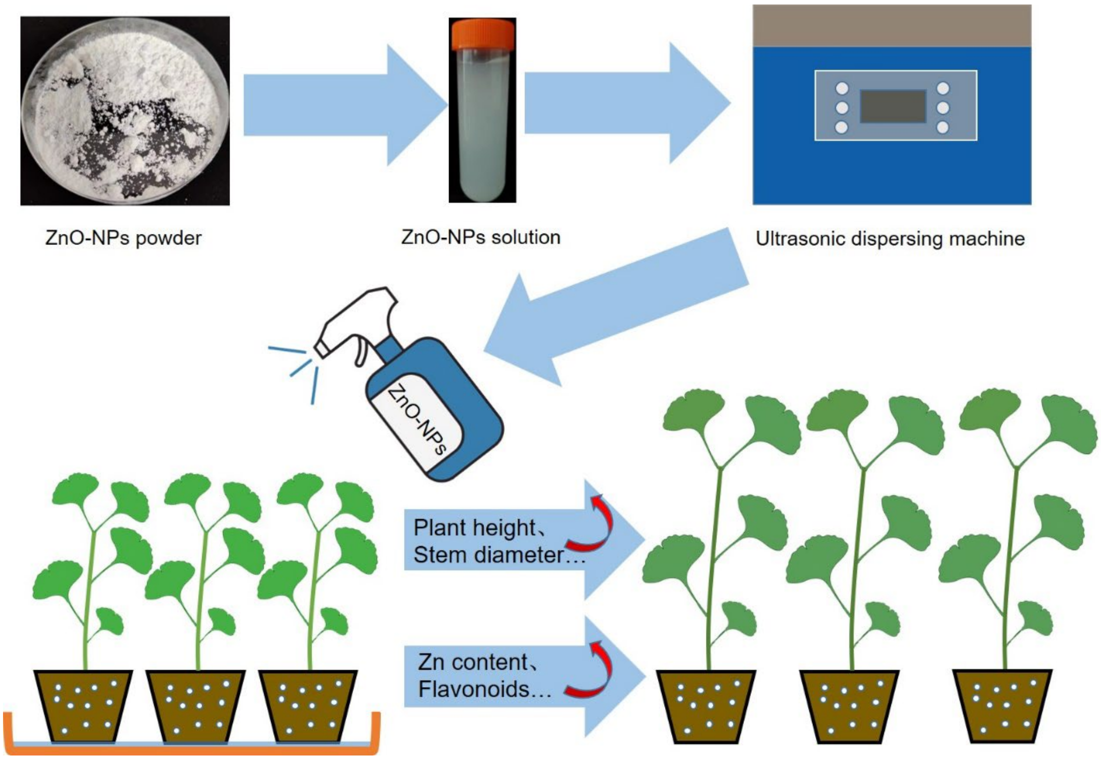

Figure S1. Simple flow chart of preparation of ZnO-NPs solution and treatment of *G. biloba* seedlings by ZnO-NPs solution.
